# The Effects of Pregnenolone 16α-Carbonitrile Dosing on Digoxin Pharmacokinetics and Intestinal Absorption in the Rat

**DOI:** 10.3390/pharmaceutics2010061

**Published:** 2010-03-15

**Authors:** Simon Lowes, Iain S. Haslam, Britt-Marie Fihn, Constanze Hilgendorf, Johan E. Karlsson, Nicholas L. Simmons, Anna-Lena Ungell

**Affiliations:** 1 Epithelial Research Group, Institute for Cell and Molecular Biosciences, Medical School, Newcastle University, NE2 4HH, UK; E-Mails: simon.lowes@doctors.org.uk (S.L.); Iain.haslam@astrazeneca.com (I.S.H.); n.l.simmons@ncl.ac.uk (N.L.S.); 2 Discovery Drug Metabolism and Pharmacokinetics, AstraZeneca Research & Development, 431 83 Mölndal, Sweden; E-Mails: Britt-Marie.Fihn@astrazeneca.com (B.-M.F.); Johan.E.Karlsson@astrazeneca.com (J.E.K.); Anna-Lena.Ungell@astrazeneca.com (A.-L.U.)

**Keywords:** ATP-binding cassette, ABCB1, P-glycoprotein, pregnane X receptor, pregnenolone 16α-carbonitrile, induction, digoxin, pharmacokinetics, disposition, intestinal transport

## Abstract

The effect of Pgp induction in rats by pregnenolone 16α-carbonitrile (PCN) (3 days, 35 mg/kg/d, p.o.) on digoxin pharmacokinetics and intestinal transport has been assessed. After intravenous or oral digoxin dosing the arterial and hepatic portal vein (oral) AUC_(0-24h)_ were significantly reduced by PCN pre-treatment. Biliary digoxin clearance increased 2-fold following PCN treatment. PCN significantly increased net digoxin secretion (2.05- and 4.5-fold respectively) in ileum and colon but not in duodenum or jejunum. This increased secretion correlated with increased Pgp protein expression in ileum and colon. Both intestinal and biliary excretion therefore contribute to altered digoxin disposition following PCN.

## 1. Introduction

The oral absorption and disposition of digoxin is markedly dependent upon the interaction it has with the ATP-dependent export pump P-glycoprotein (Pgp, ABCB1). Numerous *in vitro* systems have been used to investigate the secretory transport of digoxin by Pgp [1,2]. Because digoxin displays a very narrow therapeutic index (0.5-0.8 ng.ml^-1^; [3]), changes in intestinal secretory transport could significantly impact on efficacy or result in toxicity. Known drug-drug interactions (DDIs) effecting digoxin therapy have been highlighted by previous studies [4,5]. In man, Drescher *et al.* [5], using segmental *in vivo* perfusion of jejunum, have shown direct luminal secretion of intravenously administered digoxin sensitive to the P-glycoprotein inhibitor quinidine. Bile elimination of digoxin was, however, double that seen in the jejunum [5]. Conversely, digoxin absorption from the segmentally perfused intestinal lumen was increased by quinidine [6], showing that P-glycoprotein limits xenobiotic uptake. In addition to direct interactions with Pgp that impact on digoxin absorption, indirect DDIs have been observed involving nuclear receptor regulatory pathways. Pgp intestinal expression is not static but dynamically regulated by the pregnane X receptor (PXR) in human and rodent intestine. In man rifampacin treatment increased biliary digoxin excretion but did not significantly alter digoxin secretion across perfused jejunum [5]. Rat PXR does not respond to rifampacin [7] but pregnenolone 16α-carbonitrile (PCN) is used as an effective rodent-PXR activator [8]. In this way, Pgp induction has been confirmed in rat intestine. 

There appears to be a gradient of Pgp expression along the gut; in the rat intestine levels increase towards the colon [9,10,11]. The intestinal expression profile of Pgp is the inverse of the major Phase I metabolising enzyme, CYP3A4, which shows higher levels in the proximal regions of the gut [11]. Although this constitutive expression of Pgp has been shown to increase along the rat intestinal tract, it is not known whether PXR-mediated induction causes differential changes in functional Pgp expression in the various regions of the gut or whether the relative degree of Pgp induction is conserved throughout its entire length. 

In the context of the whole animal, it remains unclear as to the impact of increased intestinal expression of Pgp on digoxin pharmacokinetics. In the rat, digoxin pharmacokinetics are complicated by Cyp3a4-mediated hepatic metabolism, although previous studies have indicated that intestinal metabolism plays an insignificant role in digoxin disposition [12]. In the present study, both intravenous and oral digoxin pharmacokinetics following enterally adiministered PCN treatment have been investigated. Hepatic portal vein sampling provides a more direct assessment of the effects of intestinal expression on absorption. Furthermore the role of hepatic uptake and biliary excretion of digoxin in man [5] implies that enterohepatic circulation of digoxin will occur. The use of bile-cannulated animals therefore allows the direct determination of the effects of PCN pre-treatment on biliary excretion to be determined.

The overall study aims were therefore to investigate how p.o PCN activation of PXR impacts on digoxin pharmacokinetics, intestinal Pgp levels and digoxin absorption. 

## 2. Experimental Section

### 2.1. Animals

Male Sprague-Dawley rats (Harlan, Netherlands), aged approximately 100 days and weighing 250-400 g, were used in the experiments. Animal dosing and surgical/anaesthetic procedures were performed in accordance with Swedish law and ethical guidelines described in the Principles of Laboratory Animal Care (NIH publication #85-23, revised 1985).

For PCN pre-treatment, rats were dosed once daily for three days prior to experiments. PCN was given by gavage at a dose of 35 mg/kg, suspended in a vehicle of 0.1% (w/v) agarose/ water at 5 ml/kg. Bauer *et al.* [13] have shown that PCN increases Pgp expression in rat liver and brain using 3 daily divided doses at 10 and 25 mg/kg ip. Control rats were dosed with 5 ml/kg vehicle alone for the same time period.

For all surgical procedures, rats were anaesthetised using inhaled isofluorane (induction 5%, 2 L/min and thereafter 3%, 0.7 L/min) and placed on a heated table to maintain body temperature. To facilitate arterial blood sampling for pharmacokinetic measurements, all rats used in the *in vivo* studies had a polythene cannula (PP25) inserted into the left common carotid artery via a small incision made in the chest. The free end of the cannula was stoppered and passed subcutaneously out through a small incision in the dorsal nape of the neck, allowing quick access to the cannula during the experiments. For rats in which intravenous administration was required, the right external jugular vein was cannulated with polyethylene (PE50) tubing, which was again passed through the dorsal skin of the neck. In the bile collection experiments, both the left carotid artery and common bile duct were cannulated. To allow bile flow until the experiment a catheter was also inserted into the duodenum and it was also passed through the dorsal skin of the neck and connected to the bile catheter. In the portal vein studies, both the left carotid artery and the portal vein were cannulated.

### 2.2. Pharmacokinetics studies

For pharmacokinetic experiments, cannulae allowed both blood and bile sampling and did not require the animals to be anaesthetised during the procedure. Animals were dosed either intravenously with 2 MBq/kg of a [^3^H]-digoxin saline bolus (1 ml/kg over 20 seconds followed by a 150 μl flush of saline/ heparin) or orally at 4 MBq/kg (2 ml/kg by gavage). Arterial blood samples (150 µl) were taken at the appropriate time points from the carotid artery cannulae and the volume was replaced by a saline/heparin flush. Where appropriate, simultaneous samples were taken via the portal vein cannula. Bile sampling was done by allowing each bile cannula to drain continually into a glass vial throughout the duration of the experiment; at the appropriate sampling time point each was replaced by a fresh one. Blood samples were centrifuged, and 75 µl volumes of plasma or bile were counted for ^3^H activity on a scintillation counter (Wallac 1414, Finland) following correction for quench using tritiated standards prepared in rat plasma and bile. Rats were sacrificed by anaesthetic overdose following completion of the experiments.

### 2.3. Ussing Chamber studies

Rats were anaesthetised as described in the ‘Animals’ section. Following abdominal incisions, intestinal lumens were occluded by thin cotton thread proximal to the sections of tissue taken from duodenum, jejunum, ileum and colon. Sections were washed with 2 x 20 ml of ice-cold Krebs’-bicarbonate Ringer (KBR) solution (pH 7.4) (108.0mM NaCl, 4.7mM KCl, 0.6mM Na_2_HPO_4_/KH_2_PO_4_, 16mM NaHCO_3_, 1.2 mM MgSO_4_, 1.2 mM CaCl_2_, 4.9 mM Na-Pyruvate, 5.4mM Fumarate, 4.9mM L-Glutamate, 11.5mM Glucose) to remove chyme and faecal matter. The segments were placed in a chamber containing ice-cold KBR perfused with carbogen for 30 minutes. Removal by dissection of the serosal layer of the duodenal, jejunal and ileal sections and the serosa and muscularis externa of the colonic sections was performed with the aid of a stereo microscope (Wild M8) and a light source (Schott KL 1500). 

Intestinal segments were mounted as flat sheets in a modified Ussing chamber (Department of Pharmaceutical Technical Support at Astra Hässle AB). The exposed tissue surface area was 1.14 cm^2^. Agar bridges (6% Agar-Agar in 0.9% NaCl) were used to connect reference electrodes (calomel for Potential Difference, PD, (mV) measurements and Ag/AgCl for current passage). Asymmetry in the PD arising from the calomel/agar bridges were zeroed prior to tissue mounting. Likewise correction for voltage drop across the series fluid resistance upon current passage was made in assembled chambers prior to tissue mounting. 

After 30-40 minutes’ equilibration at 37^o^C, mean PD and SCC values for duodenum, jejunum and ileum of 4.1 ± 0.4, 6.4 ± 0.5, and 6.2 ± 0.5 mV (basal solution electropositive) and 161 ± 11, 271 ± 23, and 202 ± 21 μA/cm^2^ were recorded. A larger mean value of 8.1 ± 0.9 mV (basal solution electropositive) but a SCC of 94 ± 13 μA/cm^2^ for colon was observed. These values are entirely comparable with other studies [14]. 

The KBR in “donor” or “receiver” wells was replaced with 10 ml of test solution containing donor tracers of [^3^H]-digoxin and [^14^C]-mannitol or KBR to initiate the experiment. At specific timepoints, samples were remover and mixed with 5 ml of scintillation cocktail (OptiPhase ‘HiSafe’ 2 Liquid Scintillation Cocktail, Wallac) and the [^3^H] and [^14^C] activities determined using a liquid scintillation counter (Wallac, Finland).

The apparent permeability coefficients (Papp; Pa-b & Pb-a) were calculated using the equations below:

J_a-b_ = (D_b_ * M)/ (Dt *s* SA); P_a-b_ = J_a-b_ / C_a_ (1)

J_b-a_ = (D_a_ * M)/ (Dt *s * SA); P_b-a_ = J_b-a_ / C_b_ (2)

where D_a_ and D_b_ represent the activity (in dpm) transported to the apical and basal receiver compartments, respectively. D_t_ is the total activity present in the donor compartment, M is the total number of moles of substrate present in the donor compartment, s is the time in seconds, SA represents the area of tissue exposed to the solution (1.14 cm^2^) and C_a_ and C_b_ represent the concentration of the substrate in the donor compartment in moles/cm^3^. P_a-b,__b-a_ or P_app_ is expressed in cm/s.

The permeability of the inert sugar-alcohol mannitol as a paracellular flux marker [2,15] was determined by addition of the radiolabelled [^14^C]-mannitol to the pre-prepared donor solutions. 

### 2.4. Western transfer

Protein was extracted from excised sections of rat intestinal mucosa. Using a 2 mL glass homogeniser, tissue was broken down in a lysis buffer consisting of 250 μM EDTA (pH 8.0), 250 μM EGTA (pH 8.0), 320 μM sucrose, 500 μM Tris (pH 7.6), 1% Triton X (v/v) and distilled water up to a volume of 10 mL, with the addition of 1 proprietary Complete Protease Inhibitor Cocktail tablet (Roche, Cat No. 04693116001). Proteins were separated on 8% SDS-polyacrylamide gels and transferred onto PVDF membranes (millipore) overnight. Membranes were briefly washed with TBS-T (Tris-buffered saline and 0.1% Tween 20, pH 7.6) and blocked in 3% non-fat dried milk in TBS-T buffer. Membranes were probed overnight at 4 °C with the mouse monoclonal antibody C219 (Calbiochem), directed against an internal epitope of human MDR1 cross-reacting with rodent Pgp. The antibody was diluted 1:50 in TBS-T buffer. Following further washing, membranes were incubated with HRP-labeled anti-mouse IgG (AbCam) and bands detected with the pico-chemiluminescence substrate (Pierce) following manufacturers’ instructions.

Equal loading was confirmed by re-probing blotted membrane for β-actin. Membranes were stripped and washed with TBS-T. They were probed with the mouse anti-human β-actin antibody (AbCam) diluted 1:100 in 5% milk (in TBS-T buffer). Secondary staining and chemiluminescence was performed as described above. 

### 2.5. Materials

Buffer components and PCN were supplied by Sigma. [^3^H]-digoxin (specific activity 23.5 Ci/mmol) and [^14^C]-mannitol (specific activity 30 Ci/mmol) were bought from PerkinElmer. The anti-MDR1 mouse monoclonal antibody C219 was purchased from Calbiochem (Merck Chemicals Ltd, Nottingham, UK). The anti-mouse HRP conjugates from AbCam (Cambridge, UK).

### 2.6. Pharmacokinetic calculations

Pharmacokinetic evaluation was performed using WinNonlin Enterprise, version 4.1 (Pharsight Corporation, California, USA), and data are expressed as mean ± SEM. Biliary clearance (CLbile in L/h/kg) at the final time point (8 h) was calculated as follows: CL_bile_ = cumulative amount of [^3^H]-digoxin in bile (nmol)/portal vein AUC_(0-8h)_ (nmol/h/L)/body weight (kg).

### 2.7. Statistics

All data are expressed as mean ± standard error of the mean (SEM) with n representing the number of replicates in each experiment. Statistical analysis was performed using One-Way ANOVA with Bonferroni’s post-tests for multiple comparisons. Unpaired Student’s T-tests were used to compare individual pairs of experiments. All analysis performed using Prism 4 (Graphpad InStat, San Diego, California, USA). Statistical significance was reported at P < 0.05.

## 3. Results and Discussion

### 3.1. The impact of PCN-treatment on plasma [^3^H]-digoxin concentrations following oral and IV administration

**Figure 1:**
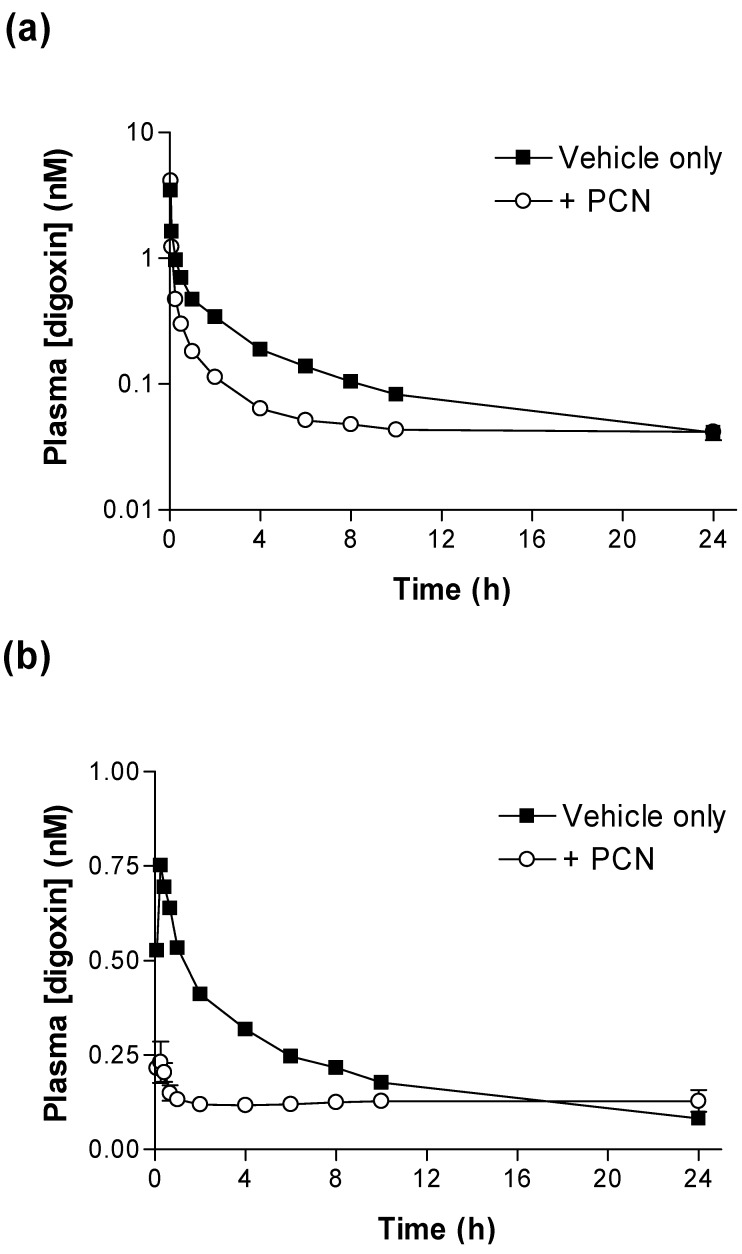
Time-dependence of [^3^H]-digoxin plasma concentrations in control and PCN treated rats following intravenous and oral administration **(a)** Arterial plasma concentrations of [^3^H]-digoxin in rats following IV administration at 2.3 nmol.kg-1 or **(b)** oral administration at 4.6 nmol.kg-1 for control (■) and PCN (○) dosed animals, treated for 72 hours prior to the experiment. Data are mean ± SEM of *n = 3* (IV) and *n = 4* (PO) animals.

Figure 1 shows that PCN pre-treatment substantially alters the pharmacokinetic plasma profiles of [^3^H]-digoxin after intravenous (IV) (2.3 nmol.kg^-1^) or oral (PO) (4.6 nmol.kg^-1^) dosing. Following IV administration (Figure 1a) the concentration of [^3^H]-digoxin remaining in blood plasma was reduced at all time points in PCN-dosed rats in comparison to control animals. The IV and oral [^3^H]-digoxin AUC_(0-24h)_ for control and PCN-treated animals are shown in Table 1. 

**Table 1:** [^3^H]-digoxin arterial and portal vein AUC in control and PCN-treated rats. Significant difference as indicated by Student’s T-test *** p < 0.001, * p < 0.05.

	Arterial AUC (nmol.hr^-1^/L)		Portal AUC (nmol.hr^-1^/L)	
Dosing	Control	PCN	Control	PCN
**IV**	3.55 ± 0.09	1.90 ± 0.03***	-	-
**Oral**	4.95 ± 0.38	2.67 ± 0.59*	3.81 ± 0.09	2.75 ± 0.09*

Significant reductions in IV and oral [^3^H]-digoxin AUC_(0-24h)_ (p < 0.001 & p < 0.05, respectively) are evident for PCN-treated animals in comparison to controls (Table 1). Following oral dosing in control animals, there was an initial rise in plasma [^3^H]-digoxin between 5 minutes (0.53 ± 0.09 nM, *n = 4*) and 15 minutes (0.75 ± 0.10 nM, *n = 4*), indicating a period of rapid intestinal uptake into the systemic circulation (Figure 1b). In the PCN-dosed animals the concentration of [^3^H]-digoxin entering the circulation is greatly reduced (Figure 1 b). This results in a reduced AUC_(0-24h)_ in the PCN-treated animals (p < 0.05 *versus* controls, Table 1) and a reduced C_max_ from 0.77 ± 0.09 nM, *n = 4* in controls to 0.27 ± 0.02 nM, *n = 4* (p < 0.005).

### 3.2. Hepatic portal vein [^3^H]-digoxin activities and biliary [^3^H]-digoxin secretion

**Figure 2:**
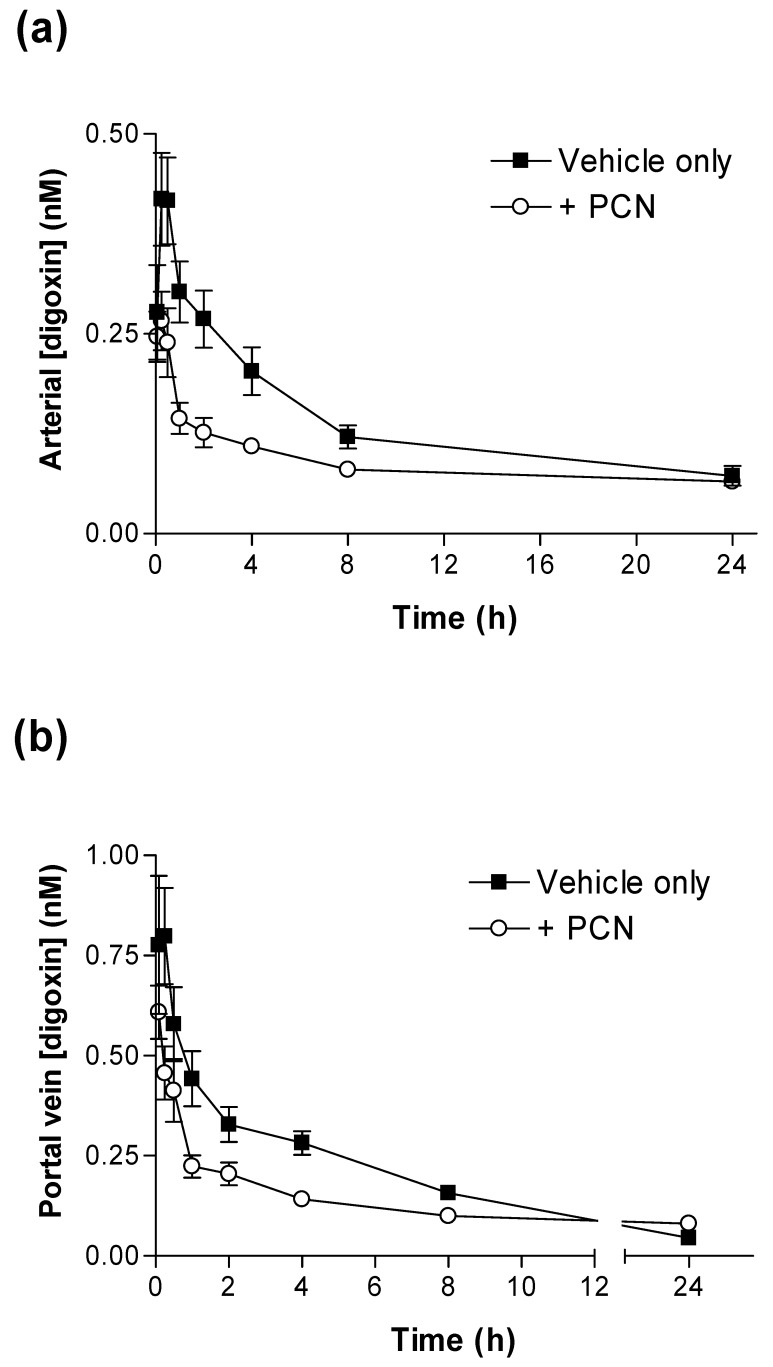
Time-dependence of [^3^H]-digoxin plasma concentrations in control and PCN treated rats following oral administration in **(a)** arterial plasma and **(b)** portal vein plasma for control (■) and PCN (○) dosed animals, treated for 72 hours prior to the experiment. Data are mean ± SEM of *n = 5* control or 6 PCN-treated animals.

**Figure 3:**
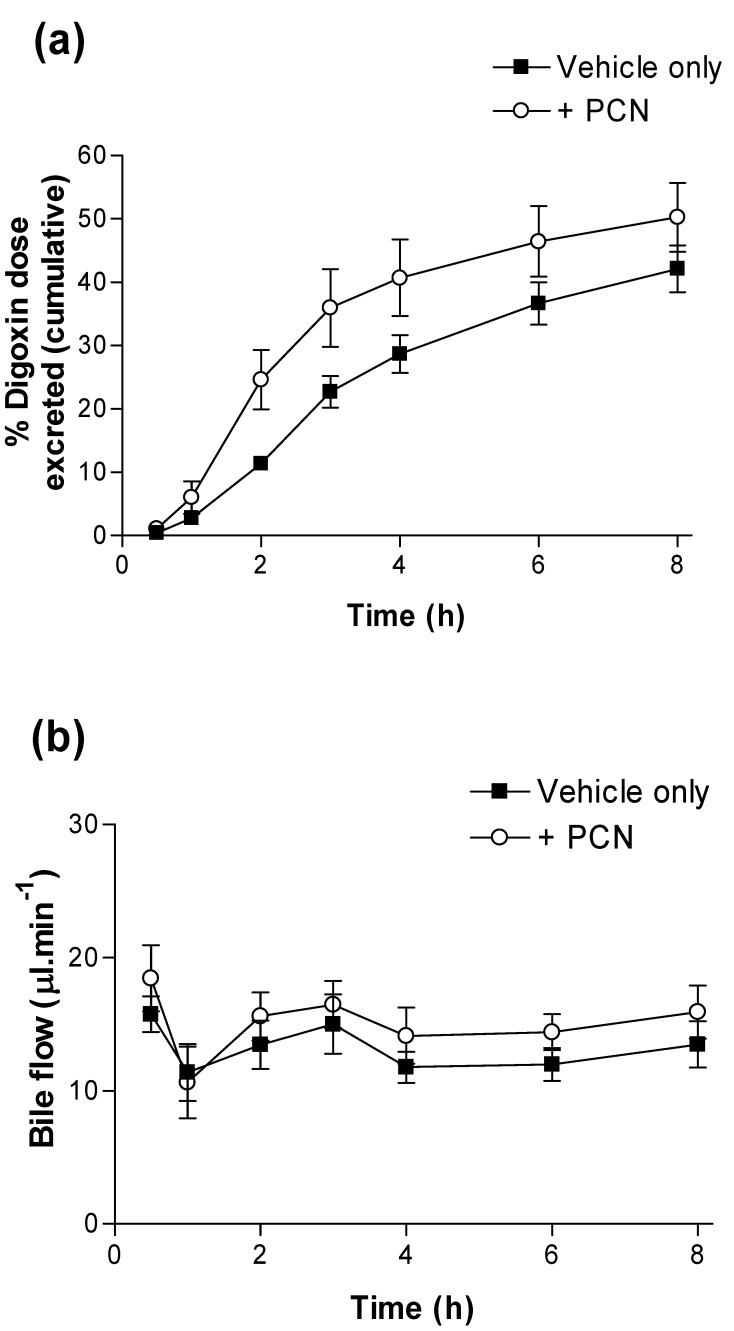
Excretion of digoxin into bile in control and PCN-dosed rats. **(a)** Cumulative dose (%) of [^3^H]-digoxin excreted into bile following oral administration (4.6 nmol.kg^-1^). Results indicate % dose in control (■) and PCN (○) dosed animals, treated for 72 hours prior to the experiment. Data are mean ± SEM of *n = 5* (control) and *n = 6* (PCN) animals. **(b)** shows the bile flow during 8 hours sampling.

In order to assess the contribution of hepatic clearance and biliary excretion of [^3^H]-digoxin/digoxin metabolites to the plasma profile following oral absorption, total [^3^H] activities in hepatic portal vein, arterial plasma and in bile were determined after oral [^3^H]-digoxin dosing (Figure 2 and Figure 3). Figure 2a confirms the initial observation that PCN pre-treatment produces a significant reduction in systemic arterial plasma [^3^H] concentrations; the AUC_(0-24h)_ in controls was 3.16 ± 0.27 nmol.hr^-1^/L (*n = 5*), which decreased to 2.04 ± 0.15 nmol.hr^-1^/L (*n = 6*) in PCN-treated animals. Figure 2b shows that in the same animals, PCN treatment also reduced portal vein plasma AUC_(0-24h)_. Portal vein plasma AUC_(0-24h)_ values are shown in Table 1, indicating a significant reduction in PCN-treated plasma concentration as compared to controls (*n = 6&*5 respectively; p < 0.02). However, portal vein C_max_ shows a non-significant reduction from 0.93 ± 0.17 nM, *n = 5* in controls to 0.62 ± 0.06 nM, *n = 6* in PCN treated animals (p < 0.1, n.s.). A significant hepatic clearance of [^3^H]-digoxin is indicated by the difference in C_max_ between control animals for portal vein and arterial plasma samples (0.93 ± 0.17 nM, to 0.44 ± 0.05 nM, *n = 5* p < 0.05) and for PCN-treated animals (0.62 ± 0.06 nM, to 0.29 ± 0.04 nM, *n = 6* p < 0.001). This data is therefore consistent with both a reduced intestinal absorption of digoxin, but also a component of enhanced hepatic extraction in the whole animal response to PCN treatment. Whereas PCN treatment decreases portal vein AUC_(0-24h)_ by 28%, arterial AUC_(0-24h)_ is reduced by 35%. Hepatic extraction reduces arterial [^3^H]-digoxin AUC_(0-24h)_ by 17% in control animals compared to 26% in PCN pre-treated rats.

Conventionally the presence of Pgp in the apical membrane of the intestine is thought to limit the epithelial absorption of Pgp substrates below the levels expected from the physicochemical properties of the compound alone (mainly lipophilicity). Increases in Pgp would therefore further limit intestinal absorptive permeability, so reducing circulating plasma levels. Hepatic portal vein blood digoxin concentrations are reduced at all but the initial time points after oral dosing, with a significant reduction in the AUC_(0-24h)_ as well as the C_max_, suggesting that *in vivo* a reduction in intestinal absorption must occur. Although digoxin is reported to be a substrate for rodent cyp3a4, studies have concluded that rat intestinal digoxin metabolism was negligible [16], with the formation of digoxigenin bis-digitoxoside (Dg2) at <1% and levels of mono-digoxitoxoside (Dg1) and digoxigenin (DG0) even lower or undetectable [12]. This metabolic profile was also found to be unaltered following PCN treatment [12]. Accordingly it is reasonable to suggest that the reduced levels of digoxin in the hepatic portal vein are the result of an increased functional Pgp expression in the intestine.

A hepatic component was confirmed by measurement of bile flow and cumulative [^3^H]-digoxin secretion via bile (Figure 3). Whereas bile flow is unaltered over the 8 hour time-course by PCN pre-treatment (Figure 3b), [^3^H]-digoxin excretion to bile is increased; at 2 hours 0.25 ± 0.05, *n = 6* of the [^3^H]-digoxin dose is excreted into bile in the PCN-treated rats compared to controls, where just0.11 ± 0.01, *n = 6* (p < 0.05) of the dose is excreted. Furthermore, the calculated CL_bile_ at 8 h rose from 0.69 L/h/kg in controls to 1.39 L/h/kg in the PCN-treated rats, a 2-fold increase.

Excretion into bile is significantly increased in the PCN treated animals at 2 hours. How is excretory transport into bile increased? Both Pgp and PXR are present in hepatocytes [17] and induction of canalicular Pgp could represent a mechanism for increased digoxin transport. However, the liver-specific oatp1a4 (old nomenclature oatp2, gene Slco1A4, previously termed Slc21A5) is a high affinity digoxin transporter which impacts on digoxin disposition [15,16] . Oatp1a4 is present on the sinusoidal membrane of hepatocytes and numerous studies have reported PXR-mediated induction of oatp1a4 in response to xenobiotics, including PCN [19,20]. An increase in hepatic sinusoidal oatp1a4 expression, coupled to similar increases in canalicular Pgp levels would explain increased biliary clearance. Increased digoxin metabolism is also possible, as PCN is also known to induce cyp3a expression [12] with hepatic metabolism by cyp3a comprising an estimated 60% of the dose [21,22,23]. Potential metabolism must therefore be taken into account when assessing whole body digoxin disposition in rat. Our attempts at separating metabolites from total [^3^H]-digoxin were unsuccessful. A similar increase in biliary secretion of ouabain, which is not metabolised, was previously observed in PCN pre-treated rats [24].

### 3.3. [^3^H]-Digoxin transport in excised intestinal sections

**Figure 4:**
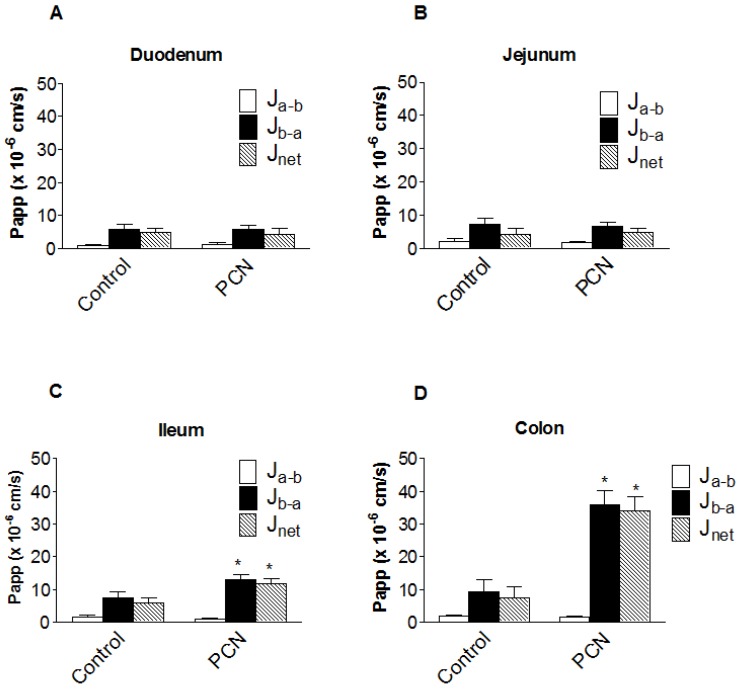
Segmental changes in intestinal bi-directional [3H]-digoxin flux in PCN-dosed rats. Transintestinal [3H]-digoxin permeability across excised sections of intestine mounted in modified Ussing chambers. Permeability was determined in both the apical-to-basal (Pa-b) and basal-to-apical (Pb-a) directions. Rats were dosed for 72 hours with an agarose vehicle control or PCN (35 mg.kg-1) prior to tissue excision. **(a)** duodenum, Data is mean ± SEM of *n* = 6 (Control) and *n* = 5 (PCN) tissue sections **(b)** jejunum, Data is mean ± SEM of *n* = 12 (Control) and n = 9 (PCN) tissue sections **(c) ** ileum, Data is mean ± SEM of *n* = 9 (Control) and n = 7 (PCN) tissue sections **(d)** colon Data is mean ± SEM of *n* = 6 (Control) and *n* = 4 (PCN) tissue sections. * P < 0.05, significantly different from control values.

From the pharmacokinetic profiles, it is apparent that intestinal digoxin absorption is substantially altered following PCN treatment. In order to assess the impact of intestinal transport on the pharmacokinetic plasma profiles, transepithelial [^3^H]-digoxin fluxes were measured across isolated intestinal sections, mounted in modified Ussing chambers (Figure 4). [^3^H]-Digoxin transport was determined to be greater in the basal-to-apical (P_b-a_) than apical-to-basal (P_a-b_) direction across all excised tissue sections (duodenum, jejunum, ileum and colon; Figure 4). Under control conditions a net [^3^H]-digoxin secretion (expressed as permeability P_net_=J_net_/C_digoxin_) of 4.88 ± 1.36 cm.s^-1^ (x 10^-6^) (*n = 6*) for duodenum, 4.20 ± 1.75 cm.s^-1^ (x 10^-6^) (*n = 12*) for jejunum, 5.79 ± 1.60 cm.s^-1^ (x 10^-6^) (*n = 9*) for ileum and 7.52 ± 3.33 cm.s^-1^ (x 10^-6^) (*n = 6*) for the colon was observed. 

The passive permeability of radiolabelled [^14^C]-mannitol was measured concurrently with [^3^H]-digoxin flux as a measure of the paracellular flux component and therefore the amount of ‘leak’ inherent within each section of intestine. Mannitol permeability was significantly different only in the colon (2.18 ± 0.40 cm.s^-1^, *n = 20*) compared to the other intestinal segments, e.g., the jejunum (6.02 ± 0.76 cm.s^-1^, *n = 43*). This difference correlates with a greater electrical resistance for colon compared to jejunum (97.9 ± 13.4 Ω.cm^2^, *n = 12* compared to 28.6 ± 1.9 Ω.cm^2^, *n = 4*3 p < 0.01). 

There were no significant changes in PD or SCC in any section of the intestine following PCN-dosing. Also for the duodenum, jejunum and ileum, PCN treatment displayed no effect on trans-intestinal resistance recordings. The resistance across sections of colon excised from PCN-dosed animals (127.6 ± 5.9 Ω.cm^-2^, *n = 9*) were slightly increased compared to controls (97.9 ± 13.4 Ω.cm^-2^, *n = 12*) p < 0.05. In addition there were also no significant changes in mannitol permeability between controls and PCN-treated duodenal, jejunal and colonic tissue segments. Overall it is apparent that PCN-dosing of animals is without effect on either tissue integrity or ion transport of the four intestinal segments used in this study.

Figure 4a and 4b show that PCN pre-treatment does not significantly alter duodenal or jejunal net [^3^H]-digoxin permeability. In the ileum however, there were significant increases in both P_b-a_ (7.43 ± 1.95 cm.s^-1^ (x 10^-6^), *n = 9* to 12.98 ± 1.51 cm.s^-1^ (x 10^-6^), *n = 7*, p < 0.05) and P_net_ (5.79 ± 1.60 cm.s^-1^ (x 10^-6^), *n = 9* to 11.89 ± 1.48 cm.s^-1^ (x 10^-6^), *n = 7*, p < 0.05) (Figure 4c). This represents a 1.75-fold increase in P_b-a_ and a 2.05-fold increase in P_net_, suggesting an increase in the functional activity of the secretory efflux transporter Pgp. A larger increase in the basal-to-apical and net permeability of [^3^H]-digoxin was seen in the excised colon sections, with P_b-a_ significantly increasing (p < 0.01) from 9.25 ± 3.77 cm.s^-1^ (x 10^-6^) (*n = 6*), to 35.93 ± 4.47 cm.s^-1^ (x 10^-6^) (*n = 4*), a 3.9-fold increase (Figure 4d). Net secretory flux also displayed the same pattern with a significant increase (p < 0.01) of 4.5-fold, from 7.52 ± 3.33 cm.s^-1^ (x 10^-6^) (*n = 6*), to 34.11 ± 4.25 cm.s^-1^ (x 10^-6^) (*n = 4*) (Figure 4d). The larger increases in colonic [^3^H]-digoxin secretion following PCN treatment suggest a greater capacity for increasing Pgp expression and activity in the distal GI tract.

Pgp activity is conventionally thought to reduce the apparent absorptive permeability of substrates when expressed in model cell-lines and in intestinal tissues [5,25]. This is most evident following Pgp inhibition (e.g. by verapamil), with an increase in the absorptive permeability (P_a-b_) of a concurrently measured substrate. Under basal conditions, [^3^H]-digoxin P_a-b_ values were 1.05 ± 0.22 cm.s^-1^ (x 10^-6^) (*n = 6*) for duodenum, 2.01 ± 0.80 cm.s^-1^ (x 10^-6^) (*n = 12*) for jejunum, 1.64 ± 0.61 cm.s^-1^ (x 10^-6^) (*n = 9*) for ileum and 1.73 ± 0.46 cm.s^-1^ (x 10^-6^) (*n = 6*) for colon. Following PCN treatment, no significant changes in [^3^H]-digoxin P_a-b_ were observed in any of the four sections (duodenum - 1.27 ± 0.51 cm.s^-1^ (x 10^-6^), *n = 5*, jejunum - 1.74 ± 0.34 cm.s^-1^ (x 10^-6^), *n = 10*, ileum - 1.08 ± 0.09 cm.s^-1^ (x 10^-6^), *n = 8*, colon – 1.48 ± 0.42 cm.s^-1^ (x 10^-6^), *n = 5*, P > 0.1). The lack of effect on P_a-b_ by the increased Pgp activity in colon and ileum mounted in Ussing chambers may indicate that *in vitro* an additional pathway for absorption of [^3^H]-digoxin is introduced by “edge effects” increasing “paracellular” diffusion above *in vivo* levels so obscuring any reduction due to Pgp. 

At the present time, there is little data on the regional expression of PXR in the intestine. PXR mRNA and protein expression is limited in proximal rat intestine relative to liver [26]. Although PXR-mediated increases in Pgp expression in human and rat intestine have been reported [12,27], these studies do not address Pgp increases along the length of the gut. As Pgp expression is reported to increase aborally along the intestine [9,28,29], Pgp-mediated digoxin efflux would be expected to be higher in the ileal and colonic segments. The present study has shown that for control rats there was only a limited increase in the magnitude of digoxin secretion (permeability) from duodenum to colon. 

Direct measurements of absorptive digoxin permeability (P_a-b_) *in vitro* after PCN treatment in all intestinal segments do not show such a reduction in P_a-b_, despite the increased net secretion of digoxin that is observed in ileum and colon. Experiments in rat brush-border intestinal vesicles have suggested that digoxin uptake is mediated by a proton-dependent carrier [30] but an additional route *in vitro* via a paracellular pathway in part arising from tissue damage may exist.

### 3.4. P-glycoprotein(Pgp) expression in rat intestinal tissue segments

**Figure 5:**
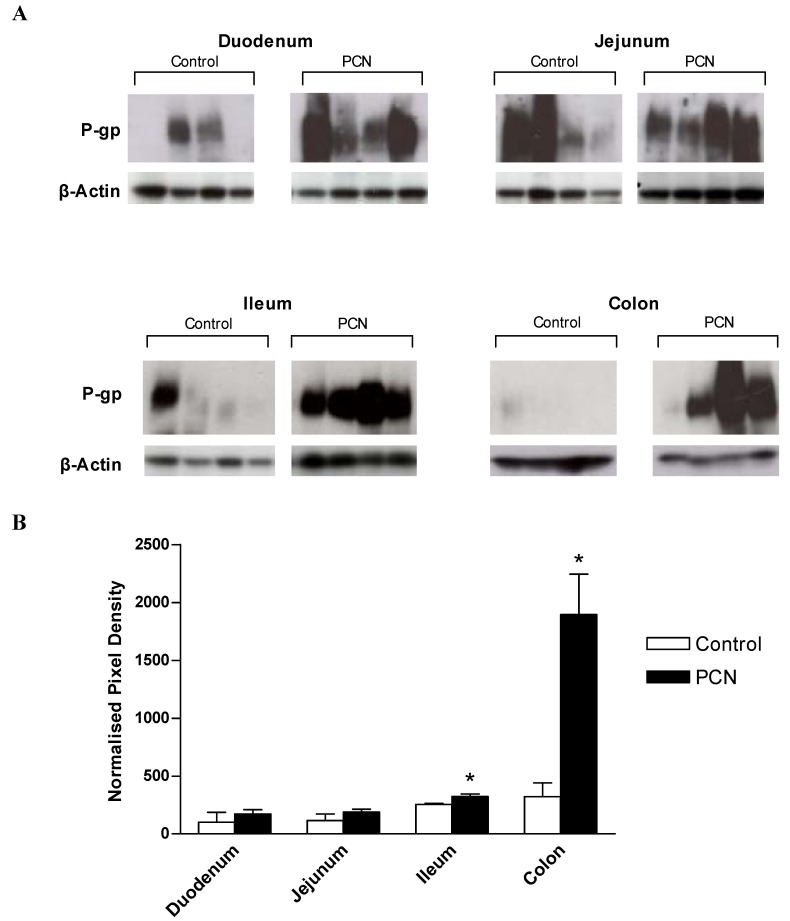
Western blot analysis of MDR1 and β-actin protein expression in excised sections of intestine from control and PCN-dosed rats **(a)** Samples of intestinal protein extracted from rat duodenum, jejunum, ileum (100 μg) and colon (50 μg) were separated by gel electrophoresis and probed for the presence of P-glycoprotein with the mouse-monoclonal antibody C219. Duodenal and jejunal blots were exposed for 5 minutes, ileal and colonic blots were exposed for 10 seconds. P-glycoprotein bands were visible at approximately 170 kDa. Blots were stripped and re-probed for the presence of β-actin with a cross-species polyclonal antibody (Abcam). β-actin bands were visible at approximately 37 kDa. **(b)** Densitometric analysis of P-glycoprotein blots indicating band pixel density relative to β-actin expression level. Data are mean ± SEM of *n = 4* protein samples. * P < 0.05, significantly different from control values.

Figure 5a shows the Western blot analysis for the expression of Pgp using the monoclonal antibody, C219. As Pgp expression levels were low in proximal intestine, chemiluminescent exposures of 5 minutes were required for duodenal and jejunal samples, whereas only a 10 second exposure was required to detect Pgp in PCN-treated ileum and colon samples. Prominent bands at approximately 170kD, representing the reported size for P-glycoprotein are evident for control samples; note that for ileal and colonic control samples, a 10 second exposure time is not sufficient to detect protein, however, longer exposure times would have led to saturation of the signal detected in the PCN-treated samples. In control samples of duodenum and jejunum there is marked variability between animals (Figure 5a). C219-stained membranes were stripped and re-probed for the expression of the constitutively expressed house-keeping gene, β-actin. Densitometric analysis of digitised images allowed expression of Pgp to be normalised to β-actin expression (Figure 5b). This data shows that whereas no significant change in the Pgp expression levels occurs in either duodenal or jejunal tissue despite the increase in mean values, both the ileum and colon display significant increases in Pgp protein levels following exposure to PCN (increases of 1.3-fold in the ileum and 2.9-fold in the colon; P < 0.05). 

Western blot analysis for control rats is therefore similar to the permeability (secretion) data in that a marginal, but non-significant increase in Pgp protein expression is seen in the distal intestine. Since Pgp expression is dynamic and dependent on dietary and environmental factors, the lack of a marked aboral gradient in either protein expression or digoxin permeability may represent the strict diet of laboratory rats not exposed to a more challenging and variable “scavenger” diet. 

Although there was a lack of variation in regional Pgp protein expression or function digoxin efflux in control animals, the present data now shows that Pgp induction potential increases aborally following 3 days PCN treatment. Net secretory digoxin transport is significantly increased in both ileal and colonic regions, with the greatest increase observed in the large intestine. This increase is matched to an increase in protein expression, with Western blot analysis indicating significant increases in Pgp protein relative to β-actin in both the ileum and colon, but with no change in the duodenum and jejunum. For the rat, secretory intestinal transport is likely limited to that mediated by Pgp [2]. That Pgp is preferentially increased in the distal intestine also implies a functional linkage between PXR and Pgp expression restricted to the distal intestine. Transit time in the upper small intestine is rapid, which is in contrast to the colonic regions where transit time is prolonged to allow for microbial fermentation and faecal dehydration by water re-absorption [31,32]. The greater increase in Pgp expression may result from an increase in both exposure time and concentration of xenobiotics, in this case PCN. Likewise, due to the reduced transit rate and increased exposure to xenobiotics, it is possible that PXR expression may be higher in colon to allow a greater capacity for increasing the physiological barrier (Pgp and CYP3A4 expression).

## 4. Conclusions

The current data has indicated that PCN treatment has a substantial impact on digoxin pharmacokinetics in the rat. Intestinal absorption is significantly reduced as a result of up-regulated Pgp expression. *In vitro* Ussing chamber data shows that the distal small intestine and colon have a greater capacity for increasing Pgp levels in response to PXR activation, which may impact on digoxin enterohepatic recirculation. Hepatic extraction is also seen to increase, partially as a result of increased biliary excretion. Our data therefore indicates a likely synergistic role for both intestinal secretion and biliary excretion in the altered disposition of digoxin in rats.
